# The Role of Necropsy in Diagnostic Dilemmas as Seen in a Tertiary Hospital in North Central Nigeria

**DOI:** 10.1155/2009/718984

**Published:** 2010-01-18

**Authors:** Olugbenga A. Silas, Adeyi A. Adoga, Agabus N. Manasseh, Godwin O. Echejoh, Barnabas M. Mandong, Rahila Olu-Silas

**Affiliations:** ^1^Department of Pathology, Jos University Teaching Hospital, PMB 2076, Jos, 930001 Plateau State, Nigeria; ^2^Department of Surgery, Jos University Teaching Hospital, PMB 2076, Jos, 930001 Plateau State, Nigeria; ^3^Ministry of Justice, Jos, 930001 Plateau State, Nigeria

## Abstract

*Background*. Necropsy (autopsy) has helped medical science and law. It has given rise to numerous diagnostic surprises as it explains cause of death, pathogenesis of diseases, and circumstances of death. It also explains reasons for most therapeutic failures. In spite of its usefulness, the rate has dropped worldwide and Africa is worse hit. This work aims to highlight the role autopsy (Necropsy) plays in demystifying diagnostic dilemmas and to encourage its patronage by medical practitioners, law enforcement agents and society. *Methods*. This is a retrospective review of autopsy and clinical reports of cases seen by pathologists and physicians in the Jos University Teaching Hospital (JUTH), Jos, North central Nigeria. *Results*. A total 166 cases were studied out of which 52 had same diagnosis for both attending physician and pathologist, 106 had different diagnoses and in eight cases diagnoses remained unknown even after autopsy was performed. *Conclusions*. Autopsy remains an important tool for obtaining definitive diagnosis, determining cause of death to explain pathogenesis of diseases, medical auditing and a vital source of data for health statistics and planning.

## 1. Introduction

Necropsy or autopsy means “seeing for yourself”. It is a systematic examination of the body after death for the purpose of not only determining the cause of death, but to explain pathogenesis of the cause of death and identifying other pathology/pathologies associated with the case [1]. 

It is used to determine the extent of a disease, the effect of treatment, and the presence of an unrecognized ailment that could have contributed to the demise of a patient [1, 2]. 

There are 2 types of autopsies, hospital and medicolegal autopsies. Medicolegal autopsy is a postmortem examination performed at the instance of the law when a coroner is instructed to determine the cause, time, and circumstance of death. Autopsies were performed in Nigeria since the early 20th century especially on European Colonial Masters [3]. Autopsy, especially medicolegal ones, was later extended to everybody in the country by 1945 [3]. In Zaria, Northern Nigeria, Edington, G.M.—a pioneer pathologist—popularized autopsy in Northern Nigeria as early as 1973 [3]. The role of autopsy in ascertaining actual cause of death in cases of antemortem diagnostic dilemma cannot be overemphasized. All over the world autopsy is been used to determine cause of death, explain pathologies of diseases, unravel circumstances surrounding death, for medical auditing and continuous medical training.

Concerning the laws of any society, autopsy has assisted in helping prosecutors in determining actual cause of death, manner of death, and circumstance of death. This has freed many innocently detained victims and helped arrest of offenders especially in homicidal cases [3].

Medical practitioners have over the years benefited from autopsy reports as it has helped to ascertain actual cause of death, evaluate therapeutic failure, explain pathogenesis of diseases, and regulate practice. 

Rare disease presentations have occasionally only been diagnosed after autopsies and patients' relatives after autopsies are better informed on cause of death. Rare diseases with genetic inheritance detected through autopsies have served as a tool for genetic counseling, screening, and monitoring of relatives. Hospital managements have used autopsy reports to identify malpractices, professional misconducts and occasionally to give statements for compensation of victims. 

Autopsy is relevant in occupational health as cause of death can determine whether victims deserve compensation or not. Many disciplinary committees in the health profession have found autopsy useful for their judgments. Many congenital anomalies have been defined in cases of stillbirths of unexplained aetiopathogenesis. This has helped genetic counseling. 

Autopsy is also a tool employed in Medicine to train doctors in Forensic Medicine. This has helped medical training, improved interest in the field of pathology as a specialty, and sharpened diagnostic acumen of medical practitioners. The numerous surprises seen from autopsy results have humbled practitioners against unnecessary heroism and unethical practices in medicine. Autopsy findings have also led to evolution of innovative diagnostic technologies to help doctors improve diagnosis and management of patients.

## 2. Materials and Methods

This retrospective study was carried out in Jos University Teaching Hospital (JUTH) Jos, North Central Nigeria.

Approval was obtained from the Ethical Clearance Committee of the Jos University Teaching Hospital.

The pathology department of this hospital has five pathologists who cater for the autopsy services of the hospital, the missionary, and numerous private hospitals in the state. They are also consulted for their service by hospitals from neighboring states of Nassarawa, Bauchi, and Benue. Data was obtained from the records section of the pathology department of JUTH. It comprised the autopsy reports and the clinical diagnosis obtained from the attending physicians. 

## 3. Results

A total of 166 cases were studied. One hundred and sixty three (98.2%) cases were medicolegal cases while only 3 (1.8%) were clinical autopsies (Table 1).

Table 2 shows the difference between antemortem and postmortem diagnosis to be different in 106 (63.9%), same in 52 (31.3%), and nil in 8 (4.8%) of the cases.

Table 3 shows examples of varying diagnosis antemortem and postmortem.

## 4. Discussion

Table 1 shows the total number of autopsies performed, medicolegal autopsies accounted for 163 (98.25) while hospital autopsies accounted for only 3 (1.8%). This shows that requests for autopsies by clinicians are very low. This is consistent with records in Europe and other parts of Africa [4, 5]. The low rate of hospital autopsies might be due to lack of skills in obtaining consent from relatives by clinicians, reluctance by clinicians to request for autopsies, advent of sophisticated diagnostic machines, fear of litigation, reluctance by clinicians to avail self for medical auditing, lack of pathologists. In Africa, sophisticated diagnostic machines are few or almost nonexistent thus making diagnosis by clinicians difficult antemortem. This calls for the need to request for autopsies for medical auditing to sharpen clinical acumen. Friedlander reported that autopsy results have been able to aid by obtaining confirmative diagnosis of patients [6]. 

Table 2 shows that in 63.9% of cases, diagnosis by pathologists after an autopsy was very different from that by clinician antemortem. This high rate is similar to that found by Friedlander and Diegbe et al. [4, 7]. The superiority of autopsy diagnosis over antemortem diagnosis was observed by Heller et al. in England [4]. 

These errors may or may not affect the survival outcome of subjects and are classified by Goldman as major or minor which may be either class I, II, III, or IV errors [8].

The Class II errors obtained in this study, which are missed major diagnosis that had no impact on survival and would not have changed therapy, were 7.5% (Table 3).

Class I errors occurred in 32 (30.2%) of the 106 cases with discrepancies in ante and post-mortem diagnosis. These missed major diagnosis had adverse effect on survival. This rate is similar to that obtained by Shojiana et al. [9]. Class III and IV errors occurred in 29 (27.3%) and 37 (35%) cases, respectively. 

The high (63.9%) percentage difference between diagnosis postmortem and antemortem clearly shows the need for more requests for autopsies by clinicians, coroners, and the general populace as many “surprises” will be observed. This will help self-medical auditing, improve diagnostic acumen of doctors, and assist in arrest of culprits.

Table 3 shows the vital role of postmortem diagnosis in identifying cause of death, mode of death, and circumstance of death. 

Clinicians should see autopsies as a welcomed source of continuous medical education and medical auditing which will generally improve the practice of medicine. It can also detect finite pitfalls in current diagnostic sophisticated medicines probably showing some of their limitations. It is also a veritable tool for explaining certain ailments and discovering associated pathogenesis hitherto not picked antemortem. Autopsy is useful also in detecting congenital anomalies in cases of unexplained stillbirths. This could be very helpful for screening and genetic counseling.

From the result it is obvious that autopsy contributed more to definitive diagnosis. This is consistent with a report by Friedlander in England who reported more than a quarter of autopsies resulting to major surprises other than the said antemortem cause of death [6]. 

In another paper by Friedlander in England titled routine natural deaths in England, he reported that in over 34% of times, the process that was believed to be cause of death prior to autopsy was completely wrong [4]. Heller et al. in England also proved autopsy diagnosis completed different but superior to antemortem diagnosis [5]. 

In Ibadan Western Nigeria, Oluwasola et al. report that though autopsy rate has generally reduced, results show its immense importance in demystifying diagnostic dilemmas [7]. Diegbe et al. in Benin Nigeria, also show role of autopsy in proper death auditing in hospitals [10]. Similar reports were observed in Zaria, Northern Nigeria by Rafindadi [11]. Mandong, Manasseh, in Jos in an earlier work on Medicolegal autopsies in North central Nigeria also showed autopsy reports on cause of death to be more useful in medicolegal cases than physician's reports especially in cases of death within 24 hours of hospital admission (coroner's cases) [12].

Obviously, coroners rely on autopsies to help prosecution [13]. It has also been used by most industries for compensation of dead workers. In hospitals, medical practitioners have benefited from autopsy reports to explain failed therapeutics, unexplained deaths, identify congenital anomalies in stillbirths. 

This has helped to sharpen their diagnostic acumen and improved training. Hospital management have also relied an autopsy reports of cause of death to determine data for rare illnesses, monitor malpractices by Medical practitioners, and obtain data for report to government for health statistics. Although the advent of sophisticated diagnosis machines has reduced autopsy rates, it still stands out as a useful procedure to explain disease pathogenesis, identifying therapeutic failures and in medicolegal cases. Public awareness on role of autopsy and its obvious use to demystify diagnostic dilemma should be emphasized. Medical practitioners should be encouraged to develop skills in receiving consent from patients and relatives for autopsy. 

## 5. Conclusions

Autopsy remains a scientific tool for verifying diagnostic dilemmas and identifying homicidal culprits. It contributes to a nation's source of data for cause of death and for health planning. Its patronage should be upheld for the good of humanity. 

## Figures and Tables

**Table 1:** Comparison of hospital (clinical) and medicolegal autopsies.

Type of autopsy	Number of cases (%)
Hospital	3 (1.8%)
Medicolegal	163 (98.2%)
**Total**	**166**

**Table 2:** Comparison of diagnosis between antemortem and postmortem reports.

Diagnosis	Number of cases (%)
Same	52 (31.3%)
Different	106 (63.9%)
Nil	8 (4.8%)
**Total**	**166**

**Table 3:** Medicolegal and hospital autopsies with their corresponding antemortem diagnosis found helpful to law enforcement agents and clinicians.

Antemortem	Postmortem
Drowning	Strangulation (homicide)
Sudden death? Cause	Cerebrovascular accident (Hemorrhagic stroke)
Typhoid fever	Bacterial meningitis
Hemorrhagic shock? Cause	Bleeding peptic ulcer disease
Multiple fractures from accidental fall from height	Strangulation (homicidal)
Disseminated tuberculosis	Meig's Syndrome
Sudden death? Cause	Ruptured Ectopic pregnancy
Typhoid Septicaemia	Viral hemorrhagic fever
